# Liver fat volume fraction measurements based on multi-material decomposition algorithm in patients with nonalcoholic fatty liver disease: the influences of blood vessel, location, and iodine contrast

**DOI:** 10.1186/s12880-024-01215-6

**Published:** 2024-02-07

**Authors:** Liuhong Zhu, Funan Wang, Heqing Wang, Jinhui Zhang, Anjie Xie, Jinkui Pei, Jianjun Zhou, Hao Liu

**Affiliations:** 1 Department of Radiology, Zhongshan Hospital (Xiamen), Fudan University, Jinhu Road No. 668, Huli District, Xiamen, Fujian China; 2 Xiamen Municipal Clinical Research Center for Medical Imaging, Xiamen, Fujian China; 3 Xiamen Radiological Control Center, Xiamen, Fujian China; 4 Department of Radiology, Zhongshan Hospital Fudan University, Fenglin Road No.180, Xuhui District, Shanghai, 200032 China

**Keywords:** Multi-material decomposition (MMD), Liver fat quantification, Fat volume fraction (FVF), Nonalcoholic fatty liver disease (NAFLD)

## Abstract

**Background:**

In recent years, spectral CT-derived liver fat quantification method named multi-material decomposition (MMD) is playing an increasingly important role as an imaging biomarker of hepatic steatosis. However, there are various measurement ways with various results among different researches, and the impact of measurement methods on the research results is unknown. The aim of this study is to evaluate the reproducibility of liver fat volume fraction (FVF) using MMD algorithm in nonalcoholic fatty liver disease (NAFLD) patients when taking blood vessel, location, and iodine contrast into account during measurement.

**Methods:**

This retrospective study was approved by the institutional ethics committee, and the requirement for informed consent was waived because of the retrospective nature of the study. 101 patients with NAFLD were enrolled in this study. Participants underwent non-contrast phase (NCP) and two-phase enhanced CT scanning (late arterial phase (LAP) and portal vein phase (PVP)) with spectral mode. Regions of interest (ROIs) were placed at right posterior lobe (RPL), right anterior lobe (RAL) and left lateral lobe (LLL) to obtain FVF values on liver fat images without and with the reference of enhanced CT images. The differences of FVF values measured under different conditions (ROI locations, with/without enhancement reference, NCP and enhanced phases) were compared. Friedman test was used to compare FVF values among three phases for each lobe, while the consistency of FVF values was assessed between each two phases using Bland–Altman analysis.

**Results:**

Significant difference was found between FVF values obtained without and with the reference of enhanced CT images. There was no significant difference about FVF values obtained from NCP images under the reference of enhanced CT images between any two lobes or among three lobes. The FVF value increased after the contrast injection, and there were significant differences in the FVF values among three scanning phases. Poor consistencies of FVF values between each two phases were found in each lobe by Bland–Altman analysis.

**Conclusion:**

MMD algorithm quantifying hepatic fat was reproducible among different lobes, while was influenced by blood vessel and iodine contrast.

## Background

Due to the increasing incidence rate of metabolic disorders, such as diabetes, obesity, and dyslipidemia [1], NAFLD has become the most common chronic liver disorder with a global prevalence of around 25% of the adult population currently [2, 3]. NAFLD refers to the presence of steatosis in more than 5% of hepatocytes in the absence of excessive alcohol consumption or other chronic liver diseases [4, 5]. It includes nonalcoholic fatty liver (NAFL) and nonalcoholic steatohepatitis (NASH). It has been recognized to have a close association with metabolic risk factors (obesity and type 2 diabetes, particularly), and is the fastest growing cause of cirrhosis, hepatocellular carcinoma [6], and cardiovascular diseases [7]. The prevalence of NAFLD-related hepatocellular carcinoma (HCC) is likely to increase concomitantly with the growing obesity epidemic globally.

Liver tissue biopsy is still the gold standard for the diagnosis of NAFLD. However, due to its invasive characteristic and poor repeatability, there is a large difference between internal and inter observer [8]. Researchers are attempting to find a noninvasive imaging way with strong repeatability to replace it. Because of the non-invasive convenience and low examination cost, ultrasound (US) is the preferred examination way for NAFLD. However, its low accuracy in detecting mild steatosis, dependence on machines and operators, low sensitivity and specificity in obese patients, obstruct its clinical application [9, 10]. Recently, the multivariable quantitative ultrasound (QUS) has been showed high diagnostic performance for detecting hepatic steatosis [11, 12], and more studies are needed to validate the result. Magnetic resonance imaging (MRI) is widely used in abdominal examinations due to its multi-parameter imaging. The fat signal in the liver can be detected and quantified by detecting proton signals using chemical shift encoding (CSE) MRI [13]. However, some factors, such as the contraindications in some patients with metal implants or claustrophobia, strict breath-hold requirement, and a bit high examination cost, obstruct its popularization in the diagnosis of NAFLD.

Until now, abdominal CT is still more commonly used than MR for routine abdominal imaging because of its appropriate exam cost, very short examination duration and independent on scanners and operators [14]. Conventionally, the degree of liver steatosis can be reflected based on the conventional CT value (HU), which is only a semi quantitative index and is considered insensitive to mild fatty liver [15]. Moreover, iron, copper and iodine in the liver may influence the attenuation of CT value. Quantitative CT (QCT) [16] can also be used in the liver fat measurement, while phantom correction and specialized software are needed, and the complex post-processing should be simplified in future. In recent years, spectral CT-derived liver fat quantification methods named MMD, which has high consistency with magnetic resonance imaging proton density fat fraction (MRI-PDFF) [17], is playing an increasingly important role as imaging biomarker of hepatic steatosis. However, there are various measurement ways with various results among different researches [18–20], and the impact of measurement methods on the research results is unknown. This study aims to evaluate the reproducibility of MMD method in NAFLD patients when taking blood vessel, location, and iodine contrast into account during measurement.

## Materials and methods

### Patients

This retrospective study was approved by the institutional ethics committee, and the requirement for informed consent was waived because of the retrospective nature of the study. Inclusion criteria: ① patients underwent non-contrast enhanced and two-phase contrast enhanced abdomen CT scanning with gemstone spectral imaging (GSI) mode on the dual-energy computed tomography (DECT) scanner; ② patients with homogeneously decreased CT density in liver when compared with that of spleen (HU_liver_/HU_spleen_ < 1), which was evaluated by radiologists. Total of 185 patients between December 2022 and June 2023 were assessed for eligibility for inclusion in the study.

The exclusion criteria were as follows: ① patients with long-term excessive intake of alcoholic and were diagnosed as alcoholic fatty liver disease (AFLD) by clinician (*n* = 16); ② patients with drug induced liver injury (DILI) confirmed by clinician (*n* = 22); ③ patients with acute pancreatitis (*n* = 5); ④ image artifacts caused by poor breath-hold or metal surrounding (*n* = 6); ⑤ patients combined with severe liver diseases such as viral hepatitis (*n* = 6) or diffuse lesions, such as liver metastasis (*n* = 23), cyst (*n* = 2) or hemangioma(*n* = 4). Finally, 101 patients diagnosed as NAFLD were enrolled in our study.

### Scanning methods

All participants underwent non-contrast phase (NCP) and two-phase enhanced abdomen CT scanning (late arterial phase (LAP) and portal vein phase (PVP)) with GSI mode on a DECT scanner (Revolution CT, GE HealthCare, Milwaukee, WI, US). Scanning parameters were as follows: dual-energy helical scanning with 80/140-kVp fast switching, tube current 315 mA; rotation time, 0.8 s; helical pitch, 0.984:1; slice thickness and interval, 1.25 mm and 1.25 mm; detector width, 40 mm; adaptive statistical iterative reconstruction-Veo (ASIR-V) of 30%. After the non-contrast enhanced data acquisition, patients were given contrast agent (Iopromide 300 mg/mL; Bayer) through a high-pressure injector at a dosage of 1.5 ml/kg body weight at a flow rate of 3.0 ml/s—3.5 ml/s. When CT value of abdominal aorta reached 220 HU with a followed-by 6 s delay time (including breath-hold guidance:3.1 s), the LAP scanning was triggered. While the PVP scanning was performed 30 s later.

### Image analysis

The data were transferred to Advanced Workstation (AW4.7; GE Healthcare), and virtual monochromatic images with energy of 70 keV and liver fat images based on multi-material composition were generated. ROIs with size of ~ 400 mm^2^ were placed at right posterior lobe (RPL), right anterior lobe (RAL) and left lateral lobe (LLL) respectively during each measurement. Measurement was carried out on the workstation independently by two experienced radiologists (Funan Wang, Heqing Wang, both had over 15 years of experience in abdomen CT), and the result was the mean value of two measurements.

ROI was firstly placed on non-contrast enhanced images (70 keV) without the reference of enhanced images and corresponding FVF was obtained, noted as FVF_without_. And then, to avoid vessels in the largest extent, ROIs were placed on the monochromatic PVP images (70 keV), on which obvious vessels were clearly shown. ROIs on LAP and NCP images were slightly placed at almost the same position with the PVP images after using the “copy and paste” function and slightly adjusting, and corresponding FVF value on NCP phase were noted as FVF_with_. The FVF value of each lobe measured on specific condition was averaged to obtain a mean FVF value.

### Statistical analysis

SPSS version 25.0 was utilized for statistical analysis. Normally distributed data was shown as mean ± standard derivation, while data with abnormal distribution was shown as median (P25, P75). The differences of FVF values between two measurements were compared using Wilcoxon matched-pairs rank test. Friedman test was used to compare FVF values among three phases for each lobe with the reference of enhanced CT images (FVF_with_: NCP vs. LAP vs. PVP), while the consistency of FVF values was assessed between each two phases using Bland–Altman analysis. Two-tailed *P* value less than 0.05 was considered as statistical significance.

## Results

The patients' characteristics are summarized in Table 1.

**Table 1:** The characteristics of patients

Characteristics			number
Total number of subjects			101
Male / Female			67 / 34
Age (mean, range)			43.6 ± 10.8 (28–72)
Purpose of enhanced CT scanning (subject number)	Follow-up of fatty liver disease (*n* = 7)		7
	Exclude suspicious masses (*n* = 21)	Abdominal pain	18
		Cirrhosis	1
		Increased tumor maker (AFP)	2
	Determine property and surroundings of masses which hinted by previous exams (*n* = 16)	Stomach mass	3
		Pancreas mass	3
		Adrenal gland mass	1
		Liver mass	3
		Lower esophageal mass	2
		Duodenum mass	4
	Excluding liver metastasis due to the history of malignant tumor (*n* = 57)	Intestinal cancer	17
		Lung cancer	13
		Breast cancer	7
		Pancreatic cancer	5
		Endometrial cancer	3
		Thymic carcinoma	3
		Esophageal cancer	2
		Other	7

### Comparison about the differences of FVF values under NCP obtained without and with the reference of enhanced CT images

**Fig. 1:**
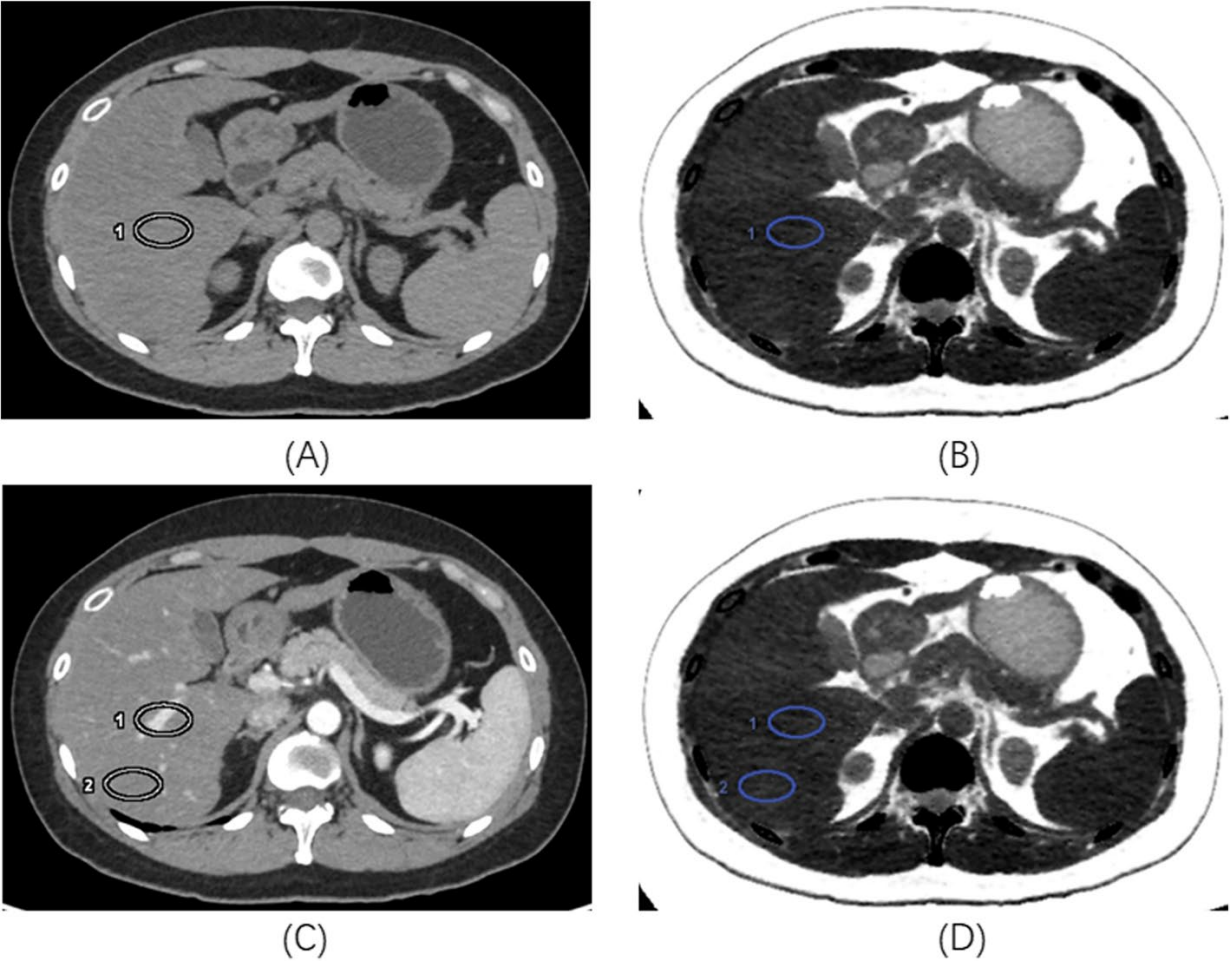
The CT images from a young man with NAFLD. A 400 mm.^2^ ROI (ROI1) was placed on the RPL on 70 keV NCP image (**A**) without the reference of enhanced CT images, and the FVF value was measured as 15.51% on the corresponding fat image (**B**). Another ROI (ROI2) was carefully placed avoiding the blood vessel according to the enhanced 70 keV PVP images (**C**), and the FVF value was measured as 16.94% on the corresponding fat image (**D**). Note: *FVF* fat volume fraction, *NAFLD* nonalcoholic fatty liver disease, *NCP* non–contrast enhanced phase, *RPL* right posterior lobe

The results showed that FVF_with_ were higher than FVF_without_ of each lobe obtained without and with the reference of enhanced CT images, as well as in mean FVF value, with all *P* < 0.05 (Table 2, Fig. 1).

**Table 2:** Comparison of FVF values under NCP obtained without and with the reference of enhanced CT images

	**Median (P25, P75)**		**Z value**	***P* value**
	**FVF**_**without**_	**FVF**_**with**_		
**RPL (*n* = 101)**	14.76 (11.21, 20.60)	15.15 (11.57, 20.49)	-2.35	0.019
**RAL (*n* = 101)**	14.98 (10.87, 19.73)	15.76 (10.91, 20.62)	-4.24	< 0.001
**LLL (*n* = 101)**	13.61 (9.15, 17.92)	14.94 (10.99, 20.57)	-6.17	< 0.001
**Mean FVF value of three lobes**	14.31 (10.35, 18.87)	15.33 (10.95, 20.00)	-6.98	< 0.001

Note: *FVF*_*without*_ fat volume fraction obtained without the reference of enhanced CT images, *FVF*_*with*_ fat volume fraction obtained with the reference of enhanced CT images, *NCP* non–contrast enhanced phase, *RPL* right posterior lobe, *RAL* right anterior lobe, *LLL* left lateral lobe

### Comparison about FVF values obtained from NCP images with the reference of enhanced CT images between each two lobes and among three lobes

The FVF values under NCP between each two lobes were obtained from NCP images with the reference of enhanced CT images. The results showed that there was no significant difference between FVFs of any two lobes (Table 2, all *P* > 0.05). The Friedman test also showed no significant differences among the FVF values of these three lobes at the same time (Table 3, *p* = 0.569).

**Table 3:** Comparison of FVF values between each two lobes and among three lobes for NCP with the reference of enhanced CT images

	**Median (P25, P75)**	**Z value**	***P* value**
**RPL vs. RAL**	15.15 (11.57, 20.49) vs. 15.76 (10.91, 20.62)	-1.03	0.305
**RPL vs. LLL**	15.15 (11.57, 20.49) vs. 14.94 (10.99, 20.57)	-0.79	0.432
**RAL vs. LLL**	15.76 (10.91, 20.62) vs. 14.94 (10.99, 20.57)	-1.65	0.099
		**χ**^**2**^** value**	***P* value**
**RPL vs. RAL vs. LLL**	/	1.129	0.569

Note: *FVF* fat volume fraction, *NAFLD* nonalcoholic fatty liver disease, *NCP* non–contrast enhanced phase, *RPL* right posterior lobe, *RAL* right anterior lobe, *LLL* left lateral lobe

### Comparison and consistency assessment about FVF values among three scanning phases for each lobe

**Fig. 2:**
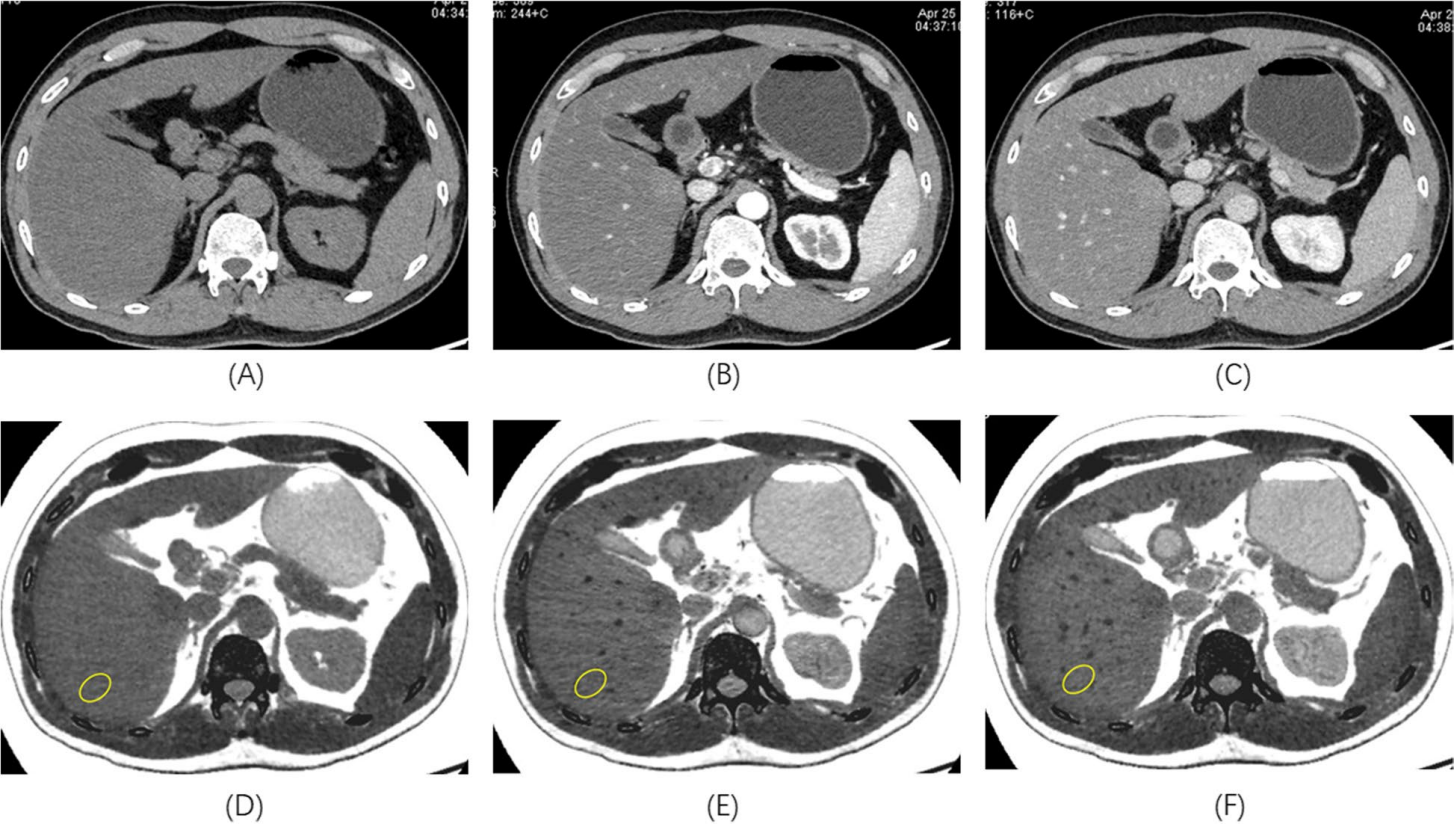
The 70 keV monochromatic images cross different scanning phases (**A**: NC, **B**: LAP, **C**: PVP) from a patient with NAFLD, and (**D**)-(**F**) were the corresponding liver fat images. Vessels could be clearly shown on PVP images (**C**), while couldnot be seen no non-contrast images (**A**). Therefore, we firstly carefully placed the ROI at RPL on PVP images avoiding vessels, and then on non-contrast and LAP images to assure the accuracy of FVF values. FVF values of ROIs (yellow ellipse) for above three scanning phases were 25.29%, 27.97%, 29.74% respectively. Note: *FVF* fat volume fraction, *NAFLD* nonalcoholic fatty liver disease, *LAP* late arterial phase, *PVP* portal vein phase, *NCP* non–contrast enhanced phase, *RPL* right posterior lobe, *RAL* right anterior lobe, *LLL* left lateral lobe

**Fig. 3:**
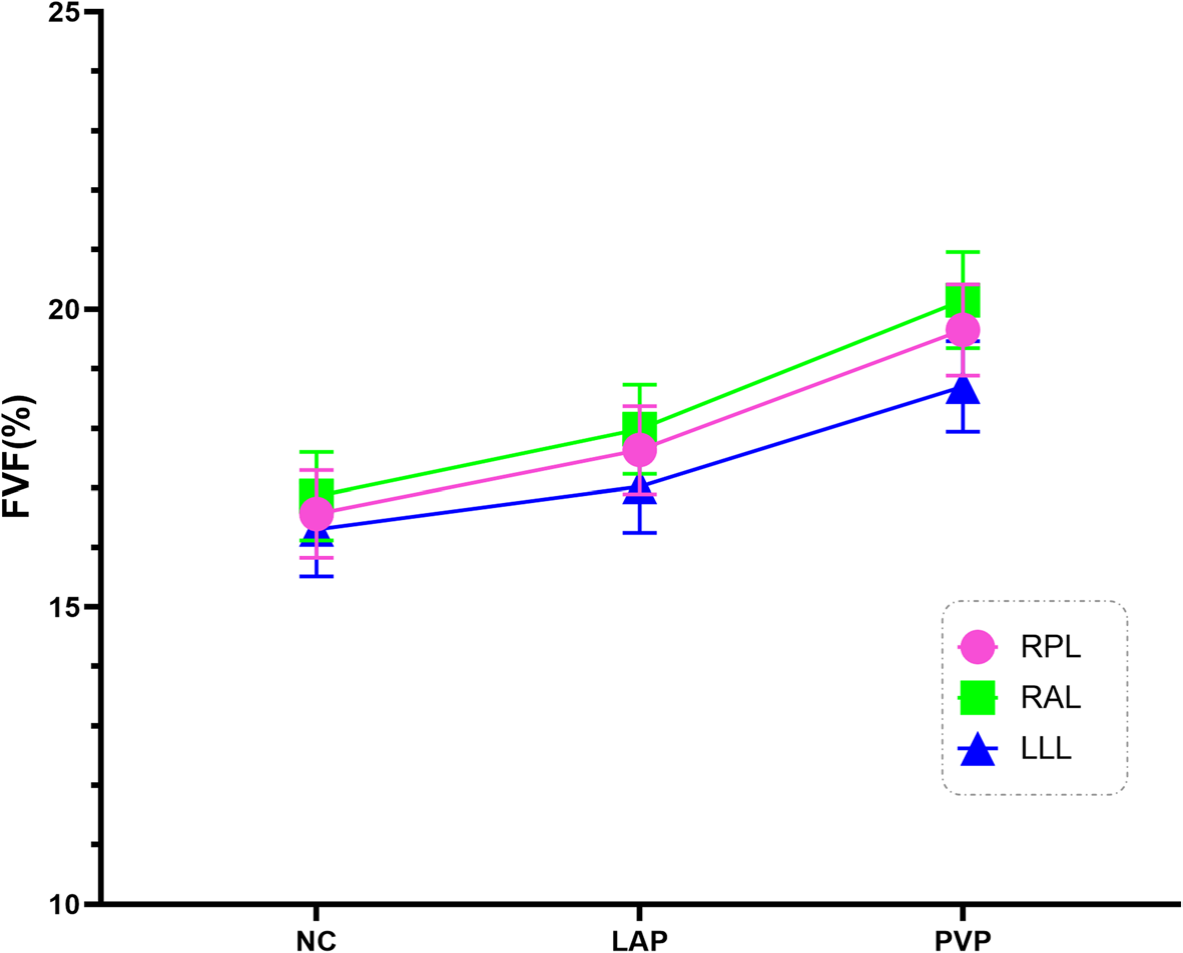
Line chart showed changes of FVF value obtained with MMD over three scan phases (NCP, LAP, PVP) in NAFLD group. Chart was colored to show the results from different liver lobes (RPL in pink, RAL in light green, LLL in blue). Means with error bars were shown. The FVF values increased after the contrast injection, and there were significantly difference among three scanning phases in the FVF values (all *p* values < 0.001 for each lobe) by Friedman test. Note: *FVF* fat volume fraction, *NAFLD* nonalcoholic fatty liver disease, *LAP* late arterial phase, *PVP* portal vein phase, *NCP* non–contrast enhanced phase, *RPL* right posterior lobe, *RAL* right anterior lobe, *LLL* left lateral lobe

**Fig. 4:**
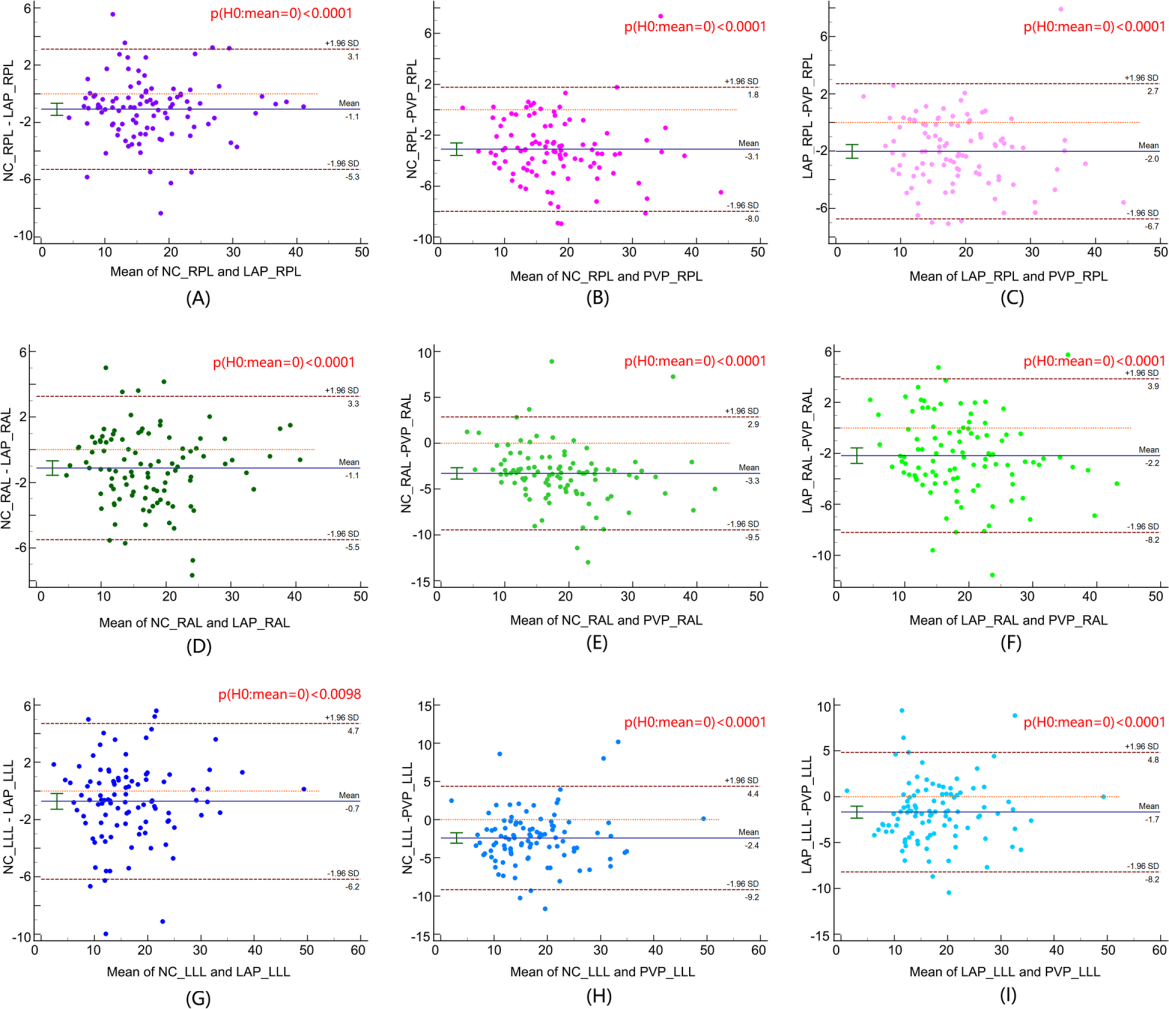
Bland–Altman plots show limits of agreement between FVF values assessed by using NCP and those assessed by using the LAP (RPL: (**A**), RAL: (**D**), LLL: (**G**), also between those assessed by using NCP and PVP (RPL: (**B**), RAL: (**E**), LLL: (**H**), as well as between those assessed by using LAP and PVP (RPL: (**C**), RAL: (**F**), LLL: (**I**) at contrast-enhanced CT in each lobe of NAFLD patients. Poor agreement of FVF values between every two phases was shown by paired sample t-test (all *p* values < 0.05). Note: *FVF* fat volume fraction, *NAFLD* nonalcoholic fatty liver disease, *LAP* late arterial phase, *PVP* portal vein phase, *NCP* non–contrast enhanced phase, *RPL* right posterior lobe, *RAL* right anterior lobe, *LLL* left lateral lobe

The result showed that the FVF value under PVP was highest, while under NCP was lowest (Table 4, Fig. 2), and there were significant differences in the FVF values among three scanning phases (Fig. 3). The consistency of FVF values between each two phases was assessed using Bland–Altman analysis, and poor consistencies were shown (Fig. 4, all *p* value < 0.05) in each lobe of NAFLD patients.

**Table 4:** Comparison of FVF values [median (P25, P75)] among three scanning phases for each lobe using Friedman test

	**NCP**	**AP**	**PVP**	**χ**^**2**^** value**	***P* value**
RPL (*n* = 101)	15.15 (11.57, 20.49)	16.33 (12.23, 21.87)	18.51(14.21, 23.48)	91.12	< 0.001
RAL (*n* = 101)	15.76 (10.91, 20.62)	15.80(12.59, 22.40)	19.60(13.75, 24.75)	77.60	< 0.001
LLL (*n* = 101)	14.94 (10.99, 20.57)	15.46(12.01, 20.86)	17.75(13.50, 22.46)	50.46	< 0.001

## Discussion

Currently, the screening of NAFLD mostly relies on hematology and US, however liver US shows relatively low sensitivity for hepatic steatosis. As a routine examination for abdominal morbidities, abdominal CT is a proper tool to evaluate NAFLD. As a novel tool in spectral CT imaging, MMD algorithm can distinguish more than two components at the same time [21], and evaluate liver fat content in volume quantitatively. Traditional CT uses attenuation value (HU) to assess liver fat content semi-quantitatively [22, 23]. With the development of dual-energy CT, dual-material decomposition (MD) with a material pair of fat and health liver tissue was used to perform quantitative assessment of the fat deposition in the liver, and good correlation with a fat percentage from the pathological analysis was reported [24]. However, it can only reflect the changes in fat concentration rather than the fat percentage. Different from above two methods, MMD involves three basic materials (fat, liver and other, such as iodinated contrast media), and can offer an intuitively quantitative assessment method for liver fat [20, 25]. The first step of MMD algorithm is obtaining virtual un-enhancement (VUE) images through replacing the volume of contrast agent in each voxel by the same volume of blood, and then fat quantification is performed by applying MMD with fat and healthy liver tissue in the material basis [26]. After the appearance of MMD, various studies were reported using MMD to calculated liver fat content. However, the method to measure FVF via MMD has not achieved agreements, as many conditions, such as ROI location and vessel interference, have not been optimized. To provide guidance for FVF measurement and obtain more accurate liver fat fraction, we evaluate the influences of liver vessel, ROI locations, and contrast enhancement during measurement.

In our study, we found that FVF values obtained with the reference of enhanced CT images and avoided vessels were significantly higher than those without avoiding vessels (Table 2). This result could be explained by the heterogeneous trait of ROI without avoiding vessels and this ROI was analyzed by MMD decomposition in voxels to obtain an average FVF. Actually, the vessel and blood voxels might not contain fat (little lipid could be ignored), resulting extremely lower fat fraction for these voxels and leading to lower average fat fraction than those with vessels avoided. All types of material decomposition including material pairs and multi-materials should be based on the actual substance. This indicated that visible vessels should be avoided during ROI measurement. For non-contrast 70 keV images which are often considered as the equivalent 120 kVp images and most widely used in abdominal diagnosis, the differentiation of vessels should be relied on the radiologists’ familiarity of anatomy. It is reported that lower keV could improve the contrast of soft tissues including vessels, however the value on non-contrast CT images is still sealed. Further study could be performed on the vessel display on lower keV non-contrast images, with possibly enhanced vessel observation, the applicated potential of MMD might be further elevated.

The influence of ROI location was also explored in our study. We chose three ROI locations, including right posterior lobe, right anterior lobe and left lateral lobe, which were less affected by heart beats or gastric contents, for each patient during measurement. With the reference of enhanced CT images, no significant difference between the FVF values obtained from NCP images of any two lobes or among all three lobes were found (Table 3). Therefore, the location of ROI might have no influence on the measurement of liver FVF. Notably, to find the influence of different liver segments on the FVF measurement, we did not enrolled patients with uneven fatty liver. In clinical practice, the uneven fatty liver was not uncommon, and the right lobe of the liver seemed to be more prone to fat deposition. The difference in fat content between different liver segments may be related to the blood supply.

Relevant researches reported the feasibility and accuracy of postcontrast DECT using MMD for quantification of the liver fat. In our study, the FVF value under PVP was highest, while under NCP was lowest (Table 3), and there were significant differences in the FVF values among three scanning phases in our study. The results were different form previous studies. Bo Yun Hur et al. used 16 rabbits to evaluate the performance of MMD algorithm in liver fat fraction calculation [27]. However, the difference of fat fractions could not be compared between the FVF value before and after contrast administration due to the different scanning modes. Zhang Q et al. [18] reported that FVF using MMD was independent of the scanning phases using GE Revolution CT, and the reason for the different results with our study might due to the greatly different sample sizes (19 vs. 101). Also, Tomoko Hyodo et al. [20] compared triple-phase contrast enhanced FVFs to determine the reproducibility of MMD under GE Discovery CT750 HD scanner. And they concluded that although the FVF under arterial and portal venous phases were larger than that from non-contrast phase, no significant difference was found in each grade respectively (grade 0: 5 patients; grade 1: 14 patients; grade 2: 11 patients; grade 3: 9 patients). However, the small sample size in each grade might affect the result.

In theory, the influence of contrast medium might be explained by the FVF calculation process. Generating VUE images was the first step, which determined the accuracy of FVF calculation afterwards. In most clinical circumstances, VUE image could be considered as an alternative to non-contrast images, resulting in reduction of radiation dose delivered to the patient [28, 29]. VUE images are derived from the decomposition of iodine including recognition and removal of attenuation responsible for iodine, however, this iodine decomposition is based on specific several materials, which reflects true tissue environment in some extent, but this material basis still shows distance from true tissue. Thus, VUE with removal of enhanced iodine in vessels make slight difference from true non-contrast images. D Olivia Popnoe et al. reported that VUE image showed significantly inferior depiction of liver parenchyma compared to true unenhanced images, and reminded us the limitation of VUE in diagnostic abdominal CT imaging [30]. Ananthakrishnan L et al. [31] also reported that the attenuation difference between true unenhanced and VUE was > 5HU in 55.6% and > 10HU in 24.8% of all measurements, while fat attenuation value on VUE image was significantly lower than that on true unenhanced image. Another comprehensive multi-manufacture research compared qualitative and quantitative metrics of virtual unenhanced (VUE) images among dual-source DECT (dsDECT), rapid kV-switching DECT (rsDECT), and dual-layer DECT (dlDECT) [32]. It revealed that in tissues with fatty content, rapid kV-switching DECT underestimated VUE attenuation. And these inter-scanner differences might be derived from technical differences among the technical implementations of DECT. Therefore, the underestimated VUE attenuation might cause overestimated FVF values when using rsDECT. In our study, the FVF values after contrast injection were indeed significantly higher than those on unenhanced images (Table 4, Fig. 2), which could be in lined with the above studies.

The study also had some limitations. Firstly, The iodine administrating rate was among 3.0 to 3.5 ml/s, which might not eliminate enhancement difference caused by various circulation among patients. Secondly, the ROI location on the lobe could not be the same during the measurement, although we had used the “copy and paste” function of the workstation. Additionally, if the ROIs were all placed close to the hepatic hilum in the extreme situation, the FVF value would be more susceptible to vascular effect, although we had placed ROIs in random in our study. Thirdly, the ROI size in our study was ~ 400mm^2^, the influence of the ROI size on the result was unknown. Fourth, gold reference (histopathology) for NAFLD was absent, which was also the inevitable flaw in most similar studies. Fifth, this study enrolled patients with liver CT density decreased (HU_liver_/HU_spleen_ < 1), which might exclude some patients with mild NAFLD. Lastly, the study hadn’t compared the results with MRI or US.

## Conclusion

MMD algorithm quantifying hepatic fat was reproducible among different lobes, while was influenced by blood vessel and iodine contrast.

## Data Availability

The datasets used and/or analyzed during the current study are available from the corresponding author on reasonable request.

## Abbreviations

FVF: Fat volume fraction
MMD: Multi-material decomposition
NAFLD: Nonalcoholic fatty liver disease
NCP: Non-contrast phase
LAP: Late arterial phase
PVP: Portal vein phase
ROI: Region of interest
RPL: Right posterior lobe
RAL: Right anterior lobe
LLL: Left lateral lobe
NAFL: Nonalcoholic fatty liver
NASH: Nonalcoholic steatohepatitis
HCC: Hepatocellular carcinoma
US: Ultrasound
MRI: Magnetic resonance imaging
CSE: Chemical shift encoding
MR-PDFF: Magnetic resonance imaging proton density fat fraction
DECT: Dual-energy computed tomography
DILI: Drug induced liver injury
GSI: Gemstone spectral imaging
ASIR-V: Adaptive statistical iterative reconstruction-Veo
MD: Dual-material decomposition
VUE: Virtual un-enhancement
dsDECT: Dual-source DECT
rsDECT: Rapid kV-switching DECT
dlDECT: Dual-layer DECT
